# Scientific rationale for developing potent RBD-based vaccines targeting COVID-19

**DOI:** 10.1038/s41541-021-00393-6

**Published:** 2021-10-28

**Authors:** Harry Kleanthous, Judith Maxwell Silverman, Karen W. Makar, In-Kyu Yoon, Nicholas Jackson, David W. Vaughn

**Affiliations:** 1 Bill and Melinda Gates Foundation, Seattle, WA USA; 2 Bill and Melinda Gates Medical Research Institute, Seattle, WA USA; 3 Coalition for Epidemic Preparedness Innovations, Greater London, UK

**Keywords:** Biological sciences, Infectious diseases

## Abstract

Vaccination of the global population against COVID-19 is a great scientific, logistical, and moral challenge. Despite the rapid development and authorization of several full-length Spike (S) protein vaccines, the global demand outweighs the current supply and there is a need for safe, potent, high-volume, affordable vaccines that can fill this gap, especially in low- and middle-income countries. Whether SARS-CoV-2 S-protein receptor-binding domain (RBD)-based vaccines could fill this gap has been debated, especially with regards to its suitability to protect against emerging viral variants of concern. Given a predominance for elicitation of neutralizing antibodies (nAbs) that target RBD following natural infection or vaccination, a key biomarker of protection, there is merit for selection of RBD as a sole vaccine immunogen. With its high-yielding production and manufacturing potential, RBD-based vaccines offer an abundance of temperature-stable doses at an affordable cost. In addition, as the RBD preferentially focuses the immune response to potent and recently recognized cross-protective determinants, this domain may be central to the development of future pan-sarbecovirus vaccines. In this study, we review the data supporting the non-inferiority of RBD as a vaccine immunogen compared to full-length S-protein vaccines with respect to humoral and cellular immune responses against both the prototype pandemic SARS-CoV-2 isolate and emerging variants of concern.

## Introduction

Unprecedented progress has been made in the design, development, production, and distribution of severe acute respiratory syndrome coronavirus 2 (SARS-CoV-2) vaccines to address the coronavirus disease 2019 (COVID-19) pandemic. COVAX, a global initiative aimed at equitable access to COVID-19 vaccines led by the World Health Organization (WHO), the Coalition for Epidemic Preparedness Innovations and Gavi, The Vaccine Alliance, has delivered over 370 million doses to 144 countries and territories (https://www.unicef.org/supply/covid-19-vaccine-market-dashboard). Despite these remarkable achievements, there are serious challenges threatening the initial target delivery of 2 billion doses in 2021, such as raw material shortages, manufacturing delays, safety signals, the emergence of multiple viral variants of concern and interest (VOC/VOI)^1,2^, and unequal procurement of vaccines by high-income countries. Ultimately, these challenges may result in a significant shortfall and delay in vaccine being made available to low- and middle-income countries (LMIC).

**Fig. 1:**
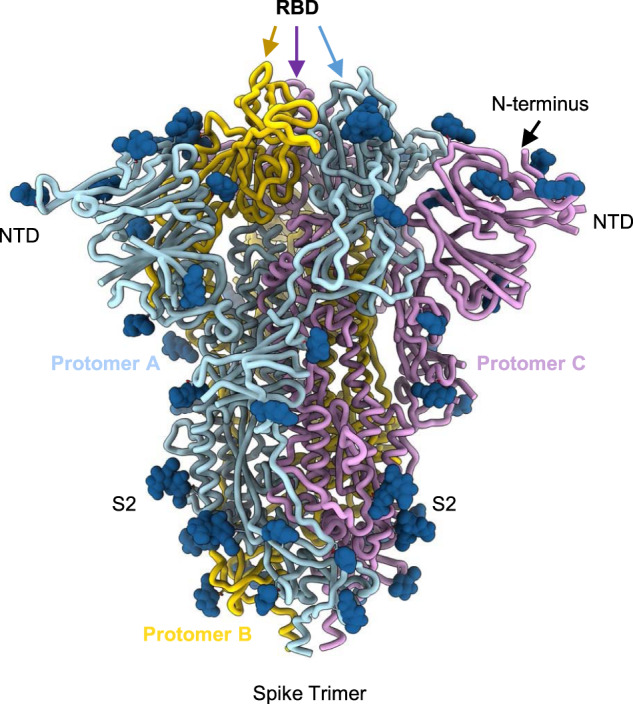
Structure of the SARS-CoV-2 S-protein in the trimeric prefusion conformation. The structure of the S-protein trimer was modeled based on PDB 7LXY^37^. The three protomers (A, B, and C) are colored in cyan, yellow, and lilac, respectively. Structural components indicated include the NTD, the RBD, the N terminus, and the S2 domain.

Most of the COVID-19 vaccines currently available or soon to be available to COVAX—including adenovirus-vectored, mRNA, and protein-subunit vaccines—use antigens based upon the SARS-CoV-2 full-length Spike (S) protein. Figure 1 illustrates the S-protein in its trimeric prefusion conformation showing the receptor-binding domain (RBD) on each protomer at the apex, the N-terminal domain (NTD) and the conserved S2 stalk region^3,4^. Vaccine approaches using just the RBD region as an antigen have the advantage of focusing immunity to key protective determinants. As an immunogen, the RBD shares multiple positive attributes with the parental full-length S-protein (Table 1), as well as potential production and manufacturing advantages such as delivery of abundant^5^, temperature-stable^6,7^ vaccine doses at an affordable cost, critical factors for distributing vaccine to LMIC. Following several successful preclinical proof-of-concept studies, RBD candidates have been advanced into clinical testing utilizing multiple vaccine platforms (Table 2). With the potential for making billions of doses of RBD vaccines available, its manufacture at single-site production facilities by experienced developing country vaccine manufacturers (DCVM) will obviate the need for numerous tech-transfers and minimize the risk of delays. This will be especially important in case there is a need to update the vaccine based on epidemiological trends, as is the case for seasonal influenza vaccines.

**Table 1:** Properties of RBD-based vaccine immunogens compared to full-length S-protein immunogens.

Properties	RBD	S-protein	Comments
Structure	3°	4°	Tertiary structure recognized by conformational nAb
Neutralizing antibody titer	High	High	>90% Target RBD but Spike protein offers anti-NTD and anti-S2 nAb
RBD epitopes	Yes	Yes	RBD epitopes appear to undergo convergent evolution and cross-protect against CoV-2 variants and other sarbecoviruses.
NTD epitopes	No	Yes	NTD is showing deletions, insertions, and divergent substitutions
Neutralizing : binding antibody ratio	High	Medium	Induction of nAb that contribute to efficacy (CoP) favors an immune-focused strategy
CD4^+^ epitopes	Medium	High	RBD-specific immunity can be augmented by multimeric display (virus-like particles) and adjuvants
CD8^+^ epitopes	Low	Low	Subunit approaches (S-protein and RBD) appear to be devoid of CD8^+^ T-cell responses. S-protein offers better coverage for cell-mediated immunity (CMI).
B-cell memory	High	High	RBD-specific memory B cells show evidence of somatic hypermutation, which increases breadth of neutralization
Antibody persistence	Yes	Yes	Demonstrated >6 months (e.g., SK bioscience RBD-np and Pfizer-BioNTech S-protein vaccines)
NHP efficacy	High	High	Proof-of-concept demonstrated against upper and lower respiratory tract infection and disease
Clinical efficacy	Yes	Yes	15 S-protein-based vaccines have reported ≥50% efficacy, with variations depending on the dominant viral variant. News reports of >90% for two Cuban RBD-based vaccines.
Supply volume	High	Medium	COVAX has negotiated favorable access terms for RBD vaccine candidates
Cost	Low	Medium	COGs low as produced by developing country vaccine manufacturers

**Table 2:** RBD-based vaccines in clinical development.

Developer and vaccine	Manufacturing platform	Antigen and adjuvant	Dosing schedule (μg RBD)	Current clinical phase	Neutralizing antibody (vaccine/HCS)	References and Clinical Trial registration number
Adimmune AdCOVID	Baculovirus	RBD^a^ + Alum		Phase 1		NCT04522089
Akston Biosciences AKS-452	CHO	RBD^a^–Fc fusion protein	1 Or 2 doses: 22.5, 45, or 90 μg	Phase 1/2		NCT04681092
Biological E RBD219-N1C1	Pichia	RBD^331–549^ + Alum/CpG	2 Doses: 10 or 25 μg	Phase 2/3	23-Fold higher in mice	^5,88,89^, CTRI/2021/06/034014
Center for Genetic Engineering and Biotechnology Abdala CIGB 66	Yeast	RBD^331–529^ + Alum	3 Doses: 50 μg	Authorized in Cuba citing 92.28% clinical efficacy	~8-Fold higher in NHP	^113,114^, RPCEC00000359
Center for Genetic Engineering and Biotechnology Mambisa CIGB 669	Yeast	RBD^331–529^ + HBV nucleocapsid	3 Doses: 50 μg (intranasal) or 2 doses in combination with CIGB 66	Phase 2		RPCEC00000345
Covaxx UB-612	CHO	RBD^340–359^-Fc + 6 peptides + CpG/Alum		Phase 2	≥50-Fold higher in guinea pig (qNeu ELISA)	^86^, NCT04773067
EuBiologics EuCorVac-19	CHO	RBD^319–541^ + MPLA lipid nanospheres	2 Doses 10 or 20 μg	Phase 1/2	~5-Fold higher in mice	^111^, NCT04783311
Finlay Vaccine Institute Soberana 02	Baculovirus	Tetanus toxoid conjugated RBD^a^-Alum	2 Doses 25 μg followed by 50 μg RBD-dimer with alum (FINLAY-FR-1A)	Authorized in Cuba citing 91.2% clinical efficacy		^115^, RPCEC00000354
Hong Kong University LAIV	Hens’ eggs	RBD^a^ (no adjuvant)	2 Doses: 5 × 10^6^– 5 × 10^7^	Phase 1		NCT04809389
Pfizer/BioNTech BNT162b1	mRNA	RBD^a^-trimer	2 Doses: 30 μg	Phase 1	4.6-Fold higher in clinical study	^74,75,82,83^, NCT04380701
Serum Institute of India HBsAg-RBD-VLP	Pichia and Hansenula	RBD-VLP^332–532^ + Alum	2 Doses: 1.8 or 2.1 μg	Phase 1/2	>50-Fold higher for NHP, 4.6-fold higher for mice	^91,101,112^, ACTRN12620000817943
SK Bioscience GBP510	CHO + *E. coli*	RBD^328–531^-nanoparticle + AS03	2 doses: 25 μg	Phase 3	5–8-Fold higher in clinical study	^26,78^, NCT04750343
Walvax/Abogen ARC0V-mRNA	mRNA	RBD^319–541^	15 μg	Phase 3		^7^, NCT04847102
West China Hospital SARS-Cov-2 RBD	Baculovirus	RBD^319–545^ + Alum	2 Doses: 40 μg	Phase 3		^87,117^, NCT04904471
Anhui Zhifei Longcom Biopharmaceutical ZF2001	CHO	RBD^319–537^ dimer + Alum	3 Doses: 25 μg	Phase 3	2-Fold higher in clinical study	^76,77^, NCT04646590

For current status of COVID-19 vaccine development, see https://covid19.trackvaccines.org/vaccines.

^a^RBD sequence data not available.

Available data are evaluated here from numerous preclinical and clinical studies, supporting the non-inferiority of RBD vaccine immunogens compared to full-length S-protein (Table 1), both with respect to eliciting homotypic and heterotypic cross-neutralizing antibody (nAb) responses and cross-protection against emerging variants.

## Clinical data support nAb as a biomarker of protection

Vaccine-induced immunity and efficacy are being used to help establish a correlate of protection (CoP), a measurable immune response (often nAb titer) predictive of protection, as has been demonstrated for some licensed vaccines^8,9^. As of October 2021, both meta-analyses and prospective case–cohort sampling analyses have found a strong correlation between nAb titers and protection against COVID-19^10–13^. This is underpinned by passive immunization and preclinical vaccine efficacy studies in nonhuman primates (NHPs) that similarly support nAb as a CoP^14–16^.

Meta-analyses of numerous clinical candidates support the correlation between vaccine efficacy and nAb titer, regardless of vaccine platform. In seeking to establish a CoP for COVID-19 vaccines, both Earle et al.^11^ and Khoury et al.^12^ initially showed only a weak relationship between efficacy and reported nAb or binding antibody (bAb) titers^11,12^. This poor association could be explained by the high variation observed between the selected assays used by different studies^17,18^. This variability was partially mitigated by calibrating the assays against panels of human convalescent sera (HCS) run within the same study. When the titers were calibrated to HCS, a strong correlation between nAb and efficacy was observed, irrespective of the specific assay platform used (rank correlation *ρ* = 0.79). nAb calibrated against HCS accounted for up to 88.4% of the variation in efficacy observed across seven different vaccine studies and suggest that any vaccine, including RBD-based vaccines, which elicit significant amounts of nAb, is likely to afford protection. The correlation was further strengthened (rank correlation *ρ* = 0.86) when the analysis uncoupled the timing of the second dose and efficacy in the AstraZeneca ChAdOx trial^13^. A recent pre-publication investigating direct quantitative comparison of immune correlates results from the AZD12222 trial and the mRNA-1273 Coronavirus Efficacy (COVE) trial shows remarkably consistent results for nAb^10^. This direct comparison was enabled by calibrating assays and reporting results against the WHO International Standard^17^, steps that are essential for comparing clinical immunological data and by now should be the standard in the field^19^. The same pre-publication also noted that 68.5% of vaccine efficacy was mediated by the day 29 cID50 titer in the mRNA-1272 COVE trial^10^. The totality of the data support neutralization titer as a potential surrogate endpoint in future clinical trials of mRNA-1273. These statistical efforts to identify a CoP for COVID-19 vaccines are being increasingly recognized by vaccine manufacturers and regulatory agencies. The UK Medicines and Healthcare Products Regulatory Agency approved a pivotal Phase 3 study design to support vaccine authorization comparing the immunogenicity of a candidate vaccine to the immunogenicity of an approved vaccine with clinical evidence of efficacy, using nAb as a biomarker of protection^20^. Additional clinical trial-specific analyses expressed in International Units are awaited, as well as assessment of the durability of this correlation over time and impact of circulating VOC.

## RBD is a key target of nAb

During the acute phase of natural infection, there is a rapid onset of protective immunity, typified by serum IgG nAb that targets RBD^21^. Functional RBD-specific responses at mucosal surfaces have also been noted, with secretory IgA shown to offer potent neutralization^22^, highlighting a preference for both systemic and mucosal responses. Depletion experiments demonstrate that 90% or more of the neutralizing activity present in the plasma of convalescent individuals is accounted for by RBD-specific nAb^23–25^. Although RBD-specific serum IgG titers were observed to wane following infection (with a half-life of 49 days), nAb titers and avidity increased over time for some individuals, consistent with affinity maturation^23^.

A second RBD depletion study of vaccinee sera from a Phase 1 clinical study, where participants were immunized with mRNA-1273, which includes a full prefusion S-protein trimer antigen, supported up to 99% of neutralizing activity to target RBD^25^. These results indicate that neutralizing activity in both natural and vaccine-induced immunity predominantly targets the RBD, even with a full spike trimer immunogen.

## RBD is a key target for potent cross-neutralizing monoclonal antibodies

**Fig. 2:**
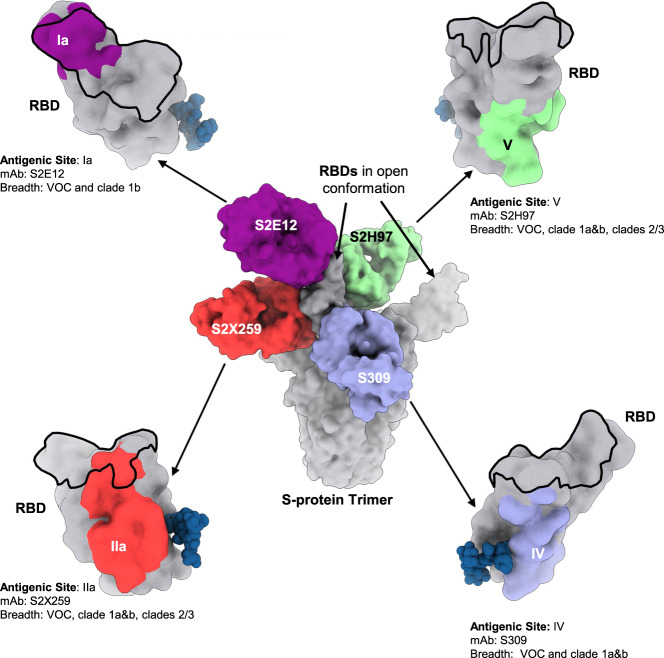
Overview of SARS-CoV-2 RBD cross-neutralizing antigenic sites. Center panel: composite model of the SARS-CoV-2 S-protein trimer (gray) with four distinct monoclonal antibodies (S2E12 in purple, S2X259 in red, S2H97 in green, and S309 in blue) bound to one RBD in the open conformation. Top and bottom, left and right panels: magnified model of RBD (gray) with the ACE2-binding site outlined in black and the respective mAb cognate epitopes indicated by color. In dark blue is a surface representation of the glycan at position N343, which is conserved across the sarbecovirus subgenus. Top left: antigenic site Ia (purple) is targeted by the S2E12 mAb, which neutralizes the VOC and clade 1b, SARS-CoV-2-related, sarbecoviruses^29^. Bottom left: antigenic site IIa (red) is targeted by the S2X259 mAb, which inhibits ACE2 binding, neutralizes the VOC and clades 1a&b SARS-CoV-2-related sarbecoviruses, and binds to clades 2/3 sarbecoviruses^30^. Bottom right: antigenic site IV (blue) is targeted by the S309 mAb, which neutralizes the VOC and clades 1a&b SARS-CoV-2-related sarbecoviruses^27,35^. Top right: antigenic site V (green) is targeted by S2H97, which neutralizes the VOC and clades 1a&b SARS-CoV-2-related sarbecoviruses, and binds to clades 2/3 sarbecoviruses^34^.

RBD exhibits tertiary structure and in its native conformation shows high affinity binding to the human ACE2 receptor (KD = 66 nM) and to conformational-dependent monoclonal antibodies (mAbs) such as CR3022 (KD = 56 nM) and S309^4,26^, a broadly cross-neutralizing sarbecoviruses mAb isolated from a convalescent patient^27^. Almost all antibodies with potent viral neutralizing activity (half maximal inhibitory concentration < 0.1 μg/ml) bind to RBD and many of them block interactions with the human ACE2 receptor^23^, which mediates viral entry into host cells (Fig. 2, ACE2-binding site of RBD outlined in black). In assessing convalescent patients, the most potent mAbs were found to bind to RBD and determined to block the RBD–ACE2 interaction at two specific sites, RBD-A (site Ia) and RBD-B (site Ib)^28–31^. Across several studies, the majority of the nAb polyclonal response (in some cases >90%) have been mapped to SARS-Cov-2 RBD, as has been the case with mAbs isolated from infected individuals^23,28,32^.

More recently, many protective epitopes have been footprinted to RBD, both within and outside of the ACE2 receptor-binding motif, with many recognized by mAbs able to cross-neutralize variants of the current pandemic, as well as other sarbecoviruses^29,30,33,34^ (Fig. 2). Of significance, is the identification of mAbs such as S309, newly identified S2H97 (determined to bind a novel RBD antigenic site designated site V), and S2E12 that bind RBD at discrete sites, and which are conserved across many clades of the sarbecoviruses^27,34^ (Fig. 2).

Of relevance to selection of the RBD as a target immunogen, the S309-neutralizing mAb recognizes an epitope containing a glycan at position N343, which is conserved across the sarbecovirus subgenus. It binds to multiple conformational states of the RBD presented from the S-protein (both open and closed) and mediates Fc-dependent effector functions such as antibody-dependent cellular cytotoxicity, supporting alternate mechanisms of protection. Its epitope resides on the opposite side of the ACE2 receptor-binding motif, which may explain its synergy with ACE2-inhibiting mAbs. S309 (evaluated in the clinic as VIR-7831) cross-neutralizes SARS-CoV-1 and SARS-CoV-2, and other sarbecoviruses^27^, and has been demonstrated to potently neutralize the Alpha (B.1.1.7), Beta (B.1.351), Gamma (P.1) and Epsilon (B.1.427/B.1.429) VOC/VOI, and protect Syrian hamsters against SARS-CoV-2 challenge in vivo^35–37^.

Based on results of a Phase 3 efficacy study, where the VIR-7831 mAb demonstrated an 85% reduction in hospitalization or death in at-risk individuals, the drug received Emergency Use Authorization from the US Food and Drug Admisnistration on 26 May 2021^38^. Potent mAbs to other RBD-specific sites have also been in clinical development (REGN10889 and LY-COVV555), although at least one has been shown to significantly lose potency against the VOC following evolutionary changes in the RBD.

To date, several RBD bAbs with potent neutralizing activity have been demonstrated to be unaffected by the RBD mutations seen in the newly circulating Alpha (B.1.1.7) and Beta (B.1.351) viral variants^29,39–41^ (for a list of the mutations in RBD, see Table 3). These anti-RBD mAbs target conserved epitopes within the RBD and are potentially attractive therapeutics based on their resistance profile and will be important for novel immunogen design.

**Table 3:** Amino acid mutations in the RBD region of SARS-CoV-2 variants of concern.

WHO Label	Pango lineage	Mutations in RBD region
Alpha	B.1.1.7	N501Y, (E484K*), (S494P*)
Beta	B.1.351, B.1.351.2, B.1.351.3	K417N, E484K, N501Y
Delta	B.1.617.2, AY.1, AY.2, AY.3	L452R, T478K, (K417N*)
Gamma	P.1, P.1.1, P.1.2	K417T, E484K, N501Y

Parentheses and * indicate the mutation is found in some sequences but not all. Information adapted from https://www.cdc.gov/coronavirus/2019-ncov/variants/variant-info.html#Concern, current as of 13 August 2021.

## NTD: a functional neutralization target

Full S-protein vaccine antigens all contain the NTD, a second neutralization target. Several NTD-specific mAbs isolated from convalescent memory B cells, as well mAbs isolated from plasmablasts following mRNA vaccination, have demonstrated potent neutralizing capabilities against the original Wuhan-1 pandemic virus^37,42^. Many of these NTD-targeting mAbs are non-neutralizing and have been shown to inhibit cell-to-cell fusion, activate alternate effector functions, and have been demonstrated to be protective from SARS-CoV-2 challenge.

The NTD is a site of prevalent and recurrent deletion regions, which vary by length and location^43^. In addition, the currently designated VOCs harbor multiple mutations in the NTD, suggesting ongoing selective pressure, with NTD highly divergent across the different variants and other sarbecoviruses^1^. As such, an S-protein immunogen risks being mismatched at the NTD site with another variant. One example is the Beta variant (B.1.351) having a deletion in the NTD, compared to the Gamma variant (P.1), which contains point mutations.

## RBD may be undergoing convergent evolution

Global sequencing efforts have documented virus evolution that includes mutations specifically in the S-protein^1,2,44^. Mutations and deletions in both the RBD (Table 3) and NTD of S-protein are of concern for ACE2 receptor-binding interactions and overall neutralizing activity^1,2^. As infectivity is reliant upon receptor binding, it has been suggested that mutation of the RBD sequence may be constrained to some degree at the ACE2-binding interface, limiting escape at this site^39^.

In support of this theory, co-evolution of separate virus lineages around the globe (Alpha: lineage B.1.1.7 or 501Y.V1 in the UK; Beta: lineage B.1.351 or 501Y.V2 in the Republic of South Africa; and Gamma: lineage P.1 or 501Y.V3 in Brazil) appear to carry one or more of the same mutations in RBD: K417N/T, E484K, and N501Y (Table 3). Similar mutations were observed to occur in forced viral evolution experiments using convalescent or vaccinee serum; passage of prototype virus with high-titer neutralizing antisera yielded a mutant virus carrying similar NTD deletions and the RBD-specific E484K mutation^45^. Mutation at the E484 site was shown to impact virus neutralization of the ancestral B.1 virus, which has been noted as a concern for emerging variants^44,46–48^ (Table 3), although recent epidemiological trends show prevalence for the Delta (B.1.617.2) variant that lacks this mutation (https://www.who.int/en/activities/tracking-SARS-CoV-2-variants/).

Many mutations in RBD appear to have enhanced receptor-binding affinity, resulting in increased infectivity and transmission. Viral variants that emerge harboring mutations in S-protein have the potential to evade nAb responses from previous infection, mAb therapies, or immunity from prior vaccination. The restricted antigenic changes observed in the SARS-COV-2 RBD support a convergent evolution theory, potentially a result of host adaptation leading to enhanced infectivity, and highlights the need for more broadly cross-neutralizing immunity^49^. As a result bivalent vaccine approaches targeting two or more variants are currently being explored for S-protein and RBD-based vaccines, in hopes of achieving broader coverage and protection against VOC^48,50^. although the utility of a bivalent vaccine formulation must be considered in the context of evolving epidemiology and additional booster shots with the prototype vaccine.

## RBD-specific memory B cells

Investigation of convalescent donor samples have shown that memory B-cell responses against the SARS-CoV-2 S-protein increase between 1 and 8 months after infection^51,52^. Analysis of convalescent donors demonstrated that RBD-specific IgG memory B-cell responses were rich in recurrent and clonally expanded antibody sequences, with 10–90% of S-protein-specific memory B cells recognizing the RBD domain^51,52^. These clonal memory B cells were determined to express antibodies with evidence of somatic hypermutation, with some having increased neutralization potency and resistance to RBD mutations. Individuals with even modest plasma-neutralizing activity have also been shown to harbor rare IgG memory B cells that produce potent SARS-CoV-2 neutralization Ab^53^. For certain antibody lineages, maturation enabled neutralization of circulating SARS-CoV-2 VOC and heterologous sarbecoviruses^54^.

Recent work has shown that vaccination is similarly able to induce robust memory. The mRNA vaccines (Pfizer BNT162b2 or Moderna mRNA-1273) efficiently primed memory B cells specific for full-length S-protein and RBD, and these were detectable in all subjects after the second vaccine dose^55^.

## CD4+ and CD8+ T-cell immune responses to RBD

Current clinical data suggest, but do not yet definitively prove, that T cells can contribute to vaccine-mediated protection against COVID-19. Providing CD4 T cell help to drive robust nAb responses is one possible mechanism of protection. It is also plausible that CD4 and CD8 T-cell responses play a larger role in modulating disease severity than in preventing asymptomatic or mild infections^56^. The role of T cells may become more pronounced in the absence of adequate nAb, such as when overall titers are low^57^, or when facing variants with mutations in nAb epitopes. For example, 50.8% vaccine efficacy was seen when nAbs were below the level of detection in the mRNA-1272 COVE trial^10^. Likewise, the Janssen Ad26.COV2.S vaccine was 52% protective in a South African clinical trial, with many cases of Beta variant infection, even though it did not elicit high antibodies against the Beta variant^58,59^. In contrast, CD8 T-cell depletion studies of convalescent primates prior to SARS-CoV-2 rechallenge only partly abrogated protection^14^ and in humans, Novavax has reported clinical efficacy of 86.3% against Alpha (B.1.1.7) and 50% against Beta (B.1.351) variants in the absence of any prominent CD8+ responses^60–62^. Sinovac^63–65^, Sinopharm^66^, and Bharat^67^ have also reported clinical efficacy without eliciting notable CD8+ responses. Thus, CD8 T-cell responses may be beneficial but are not absolutely required for protection.

Due to the anticipated benefit of vaccine-induced T-cell immunity to SARS-COV-2, CD4 and CD8 T-cell epitope mapping using overlapping peptides has been performed across the full length of the S-protein. An evaluation of CD4 T-cell responses in humans reported a frequency of at least 20% focused on discrete regions of the S-protein, including the NTD, the C-terminal domain, the neighboring fusion protein region, and the RBD^68,69^. It was also reported that the sequences of most T-cell epitopes are unaffected by mutations in the SARS-COV-2 VOC, including the Beta (B.1.351) variant^70^. Consistent with this finding, CD4 and CD8 T-cell reactivity to S-protein in vaccinees were robust against the SARS-COV-2 variant mutations.

The RBD contains fewer immunodominant CD4 T-cell epitopes than the full-length S-protein. Despite this fact, there is ample evidence that sufficient numbers of T-cell epitopes are presented by the RBD to generate good CD4 responses and elevated levels of functionally nAb^16,71^. The identification of multiple common CD4 T-cell epitopes within the RBD has been independently verified using different T-cell epitope mapping approaches^69,72,73^. Clinical studies with RBD antigens support the assertion that sufficient T-cell help is provided following immunization (Table 2). Immunization with an mRNA-encoded RBD vaccine candidate showed the induction of nAb titers ranging from 0.7-fold (1 μg dose) to 4.6-fold (30 μg dose) higher than HCS^74,75^. Notably, induction of robust nAb titers by RBD was not specific to the mRNA platform. A protein-subunit RBD-dimer vaccine co-administered with Alum, was similarly able to drive nAb responses in humans^76,77^, as was an RBD array displayed from a synthetic nanoparticle vaccine adjuvanted with AS03^78^. In summary, data on immune responses elicited by infection and vaccination with a S-protein or RBD antigen provide evidence that a potent RBD vaccine will recruit ample T-cell help.

CD8 T-cell epitopes may be less critical in subunit vaccines that protect primarily via humoral immunity. Epitope mapping of CD8 T-cell reactivity did not reveal an immunodominant region in S-protein, with epitopes being roughly equally distributed along the sequence^68^. Similar to CD4 epitopes, by definition, the RBD subdomain of S-protein contains fewer CD8 T-cell epitopes than the full-length S-protein. Therefore, it is not clear whether RBD would contain adequate CD8 T-cell epitopes for a vaccine whose protection was based on eliciting CD8 T cells. In this context, the broader number of epitopes in the full S-protein may be advantageous.

Due to large variations in T-cell assay methodology and resultant data, cross-study comparisons of T-cell responses are difficult, if not impossible, to interpret^79,80^. Full appreciation of the effect of vaccine platform and antigen on CD4 and CD8 T-cell responses will require standardized peripheral blood mononuclear cell collection and assay protocols, as was established within the COVID-19 Prevention Network and the COVE trial. Interpretable comparison of vaccine-induced cell-mediated immunity is expected within the year.

## Preclinical studies support RBD as a potent vaccine antigen

Preclinical studies have evaluated the protective efficacy of several RBD-specific monoclonals, as well as RBD as a protective immunogen. Investigators evaluating RBD as a vaccine candidate have delivered the antigen either as DNA^81^, mRNA^7,74,82–85^, viral vector^86^, soluble monomer or dimer^6,22,87–93^, a fusion protein nanoparticle^16,26,94–98^, or as a virus-like particle (VLP)^91,99–102^. Additional RBD-based vaccine candidates in clinical trials (Table 2) have not yet published their preclinical work. The RBD sequences selected from the S-glycoprotein as candidate vaccines encode an ~25 kDa (>200 aa) glycosylated protein^103^, produced using either mammalian^26^, baculovirus-infected insect cells^92^, or yeast-based production platforms^5,88,89,91,104^. In general, vaccination strategies have focused on parenteral immunization to elicit high-titer serum nAb, although some preclinical studies support intranasal delivery of S-protein in NHPs and hamsters using a live vector approach^105^, and RBD on chitosan nanoparticles in mice^106^, with evidence for a role of mucosal immunity in reducing viral load in both the upper and lower respiratory tract.

## Prototype RBD-np vaccine

The Institute for Protein Design (University of Washington) SARS-CoV-2 vaccine consists of a self-assembling, two-component nanoparticle that displays 60 copies of RBD per nanoparticle^26^. The RBD-np (nanoparticle) is produced in mammalian cells and maintains the two functional N-linked glycosylation sites (N331 and N343), important for proper folding of protein and for antibody recognition^26^. A comparison of the glycosylation patterns between the RBD-np and the S-2P trimer was very similar at the two sites, both exhibiting complex glycan that were heavily fucosylated. The RBD-np candidate administered intramuscularly proved safe, was as immunogenic as a recently described next-generation prefusion S-protein trimer^107^, and protected mice and rhesus macaques from live virus challenge. SK bioscience is developing the RBD-np vaccine technology from the University of Washington and is currently in Phase III clinical testing of the prototype variant B.1 vaccine (GPB510) adjuvanted with AS03^78^.

In initial mouse studies, the RBD-np vaccine was shown to induce nAb titers tenfold higher than the prefusion-stabilized S-protein trimer (S-2P) despite a fivefold lower dose^26^. The RBD-np vaccine induced antibodies targeting multiple distinct epitopes on the RBD, elicited a higher nAb : bAb ratio than HCS, and was fully protective against challenge with mouse-adapted SARS-CoV-2. Immunization of humanized mice transgenic for the non-rearranged human antibody variable and constant region germline repertoire confirmed the ability of this candidate to mount functional humanized nAbs, superior to that observed using S-2P prefusion trimer^26^.

NHP studies compared adjuvants (head-to-head) for advancement of the leading formulations for clinical development. Groups of rhesus macaques were immunized twice with RBD-np, plus one of five adjuvants including AS03, Alum-CpG, Alum, AS37, and another oil-in-water emulsion^16^. Both pseudovirus and live virus neutralization assays against the B.1 virus supported elevated titers with the AS03, Alum-CpG, and Alum adjuvants. Following intranasal/intratracheal challenge, significant protection was observed against viral burden in both the upper respiratory tract (URT) and in the lungs using an established sub-genomic mRNA assay with E-gene-specific primers, as well as protection against disease using positron emission tomography–computed tomography. Vaccination through intramuscular administration achieved a significant reduction in viral burden in the URT, potentially supporting the ability of RBD-based vaccines to also limit transmission, with evidence that greater serum nAb titers is inversely correlated with protection in the upper airways.

Animals similarly immunized with RBD-np adjuvanted with AS03 were followed for 5 months as part of a durability study and determined to maintain neutralizing titers (>1 : 1000) and ACE2-blocking activity^16^. The RBD-np-specific plasmablast response was also measured, 4 days after the second immunization, with the magnitude of antigen-specific IgG-secreting cells in blood correlating with the observed nAb responses. Immunized macaques exhibited an RBD-specific cell-mediated immune response, which was dominated by IL-2 and/or tumor necrosis factor-α (TNF-α)-secreting CD4+ T cells, an apparent Th1/Th2-balanced response, with little evidence of a CD8+ T-cell response.

A correlates analysis supported nAb, both using a wild-type and pseudovirus assay, as the top statistically significant CoP in both the nasal and pharyngeal compartments. Nanoparticle-specific IL2+ and TNF+ CD4 T-cell responses also emerged as a statistically significant CoP, suggesting the nanoparticle itself may contribute T-cell help^16^.

In a follow-on NHP study^16^, the RBD-np adjuvanted with AS03 was compared to the stable prefusion S-protein trimer HexaPro, where an additional four (4) Proline substitutions have been introduced to increase thermal stability and yield of production in mammalian cells^107^. Their evaluation, both as a soluble protein and displayed separately on a similar nanoparticle, supported near-equivalent neutralization titers after two doses.

Analysis of cross-neutralizing activity against VOC with sera from NHPs immunized with RBD-np adjuvanted with AS03 or Alum supported potent cross-neutralization against the Alpha (B.1.1.7) variant and favored AS03 as a preferred adjuvant for neutralization of the Beta (B.1.351) virus^16^. In pseudovirus assays, RBD cross-neutralization titers were equivalent to HexaPro immune sera for both VOC assessed, whereas RBD-np elicited higher titers of Abs against all RBD antigenic sites evaluated compared to HexaPro immune sera^97^. In addition, the data suggest that epitopes outside of the RBD do not significantly contribute to neutralization of VOC. Another NHP study evaluated the contribution of a third dose of RBD-np adjuvanted with Addavax. Primates boosted 6 months after primary immunization further elevated their cross-nAb titers against the Alpha (B.1.117) and Beta (B.1.351) variants, with GMT titers > 4e3 in a pseudovirus assay^97^.

Vaccine researchers around the world have demonstrated preclinical immunogenicity of multimeric RBD vaccine candidates utilizing a variety of presentation methods including the following: ferritin nanoparticles^94,96,98,108,109^, single-component protein nanoparticles^95^, two-component protein nanoparticles either self-assembling^26^ or assembled via SpyTag/Catcher technology^91,99,110^, and VLPs^102,111^. Together, these studies support the assertion that RBD displayed on a particle and co-administered with a suitable adjuvant represents a viable vaccine strategy for SARS-CoV-2, including against VOC.

## RBD-based vaccines in the clinic

RBD as an immunogen has been advanced to the clinic independently by many developers (Table 2). Some large vaccine manufacturers, Serum Institute of India^112^, Biological E^5^, and SK Bioscience^16,26^, are developing RBD candidate vaccines scalable to hundreds of millions of doses at single-site production facilities, obviating the need for tech-transfer.

To date, two RBD vaccines developed in Cuba have reported favorable clinical efficacy data via news outlets^113^. The Center for Genetic Engineering and Biotechnology announced 92.28% efficacy following three doses of its Abdala vaccine^114^ and the Finlay Vaccine Institute reported 62% efficacy after two doses of its Soberana 02 vaccine and 91.2% efficacy following a third heterologous boost with the RBD-dimer vaccine^115^. Scientific reports of the efficacy studies are anticipated shortly. Three additional RBD vaccines have reported positive clinical immunogenicity and compared performance to a panel of HCS for benchmarking purposes: the SK Bioscience protein-subunit RBD-np^78^, the Zhifei Longcom protein-subunit RBD-dimer with Alum vaccine^76^, and the BioNTech mRNA RBD-trimer BNT162b1 vaccine^74,75,82,83^. In a press release, SK Bioscience reported interim results of a Phase 1/2 clinical trial, stating that the GBP510 vaccine, a two-component nanoparticle displaying 60 copies of RBD adjuvanted with AS03, was safe, well-tolerated, and demonstrated 100% nAb seroconversion with GMTs five to eight times higher than that in HCS after two doses^78^. Following three doses of the Zhifei RBD-dimer ZF2001 vaccine, 97% of recipients seroconverted with nAb GMTs twofold higher than the HCS panel tested^76^. In addition, Zhifei have reported a limited reduction in neutralization (1.6-fold) against the Beta (B.1.351) variant^77^.

BioNTech and Pfizer evaluated four mRNA candidate vaccines in Phase 1/2 clinical trials, including a lipid nanoparticle-formulated nucleoside-modified mRNA that encoded the RBD fused to a T4 fibritin-derived foldon, resulting in a trimeric RBD, BNT162b1^74,75,82,83^. BNT162b1 elicited robust CD4+ and CD8+ T-cell responses, and strong humoral immunity after two doses (ranging from 1 to 50 μg per dose), with levels above that observed for HCS (1.9-fold higher at 10 μg dose; 4.6-fold higher at 30 μg dose) and equivalent to responses to the full S-protein candidate, BNT162b2^74,75,82,83^. The BNT162b2 candidate vaccine, transcribing full-length S-protein mRNA, was associated with a lower incidence and severity of systemic reactions, particularly in older adults, and therefore the RBD vaccine (BNT162b1) did not move forward clinically. The authors speculated that the difference in observed reactogenicity might be explained by the number of RNA molecules in 30 μg of BNT162b1 being approximately five times as high as that in 30 μg of BNT162b2^75^.

When we apply the ratio of the vaccine-induced vs. convalescent sera-neutralizing titer from these three RBD vaccines to the correlate analyses^10–12^, the predicted vaccine efficacy against the ancestral virus is well above 90% for both the SK Bioscience GPB510 and the BioNTech/Pfizer BNT162b1 RBD vaccines, and ~90% for Zhifei’s ZF2001.

The ability of RBD-based vaccines to impact viral burden in the upper airways and limit community transmission is still to be studied. As for S-protein-based vaccines already deployed^116^, it is likely that as immunity declines over time and variants arise, vaccinated individuals may be more likely to become transiently infected and transmit to others. This puts a spotlight on the required induction and duration of elevated nAb responses to maximize protection.

## Conclusions

SARS-CoV-2-related data from multiple vaccine researchers support nAb as the key biomarker of protection irrespective of vaccine platform, as evaluated in mice, hamsters, NHPs, and humans. The RBD of the S-protein displays the major functionally neutralizing epitopes and, when used as an immunogen, may preferentially focus the immune response to highly potent and cross-protective determinants. Isolated mAbs to these protective determinants have been shown to cross-neutralize the VOC and support selection of RBD as a pan-sarbecovirus vaccine target^49,110^. Nonclinical and clinical vaccine studies demonstrate the exceptional properties and non-inferiority of RBD as an immunogen compared to full-length S-protein (Table 1). Focusing immunity to RBD through targeted vaccination strategies may have an important impact on eliciting elevated cross-nAb titers, with subsequent protection against viral variants and potentially limiting community transmission.

Given (1) the potential manufacturing and cost advantages of RBD as a vaccine immunogen across different platform technologies^5,112^, and (2) the engagement of experienced DCVM partners that can deliver billions of vaccine doses, RBD vaccine candidates with non-inferior safety and immunogenicity should continue to be evaluated, to address a critical medical need and ensure equitable access of vaccine.

With waning immunity and regulatory discussions on potential booster doses, we speculate that the availability of an RBD-based vaccine strategy that targets immunity to key cross-protective determinants may be a considerable advantage, a vaccination strategy potentially for both the previously immunized and those naturally infected.

## Data Availability

No data were generated for the review article.
